# Characteristics of dental note taking: a material based themed analysis of Swedish dental students

**DOI:** 10.1186/s12909-020-02441-6

**Published:** 2020-12-16

**Authors:** Viveca Lindberg, Sofia Louca Jounger, Maria Christidis, Nikolaos Christidis

**Affiliations:** 1 Department of Humanities and Social Studies Education, Stockholm University, SE-106 91 Stockholm, Sweden; 2 Division of Oral Diagnostics and Rehabilitation, Department of Dental Medicine, Karolinska Institutet and Scandinavian Center for Orofacial Neurosciences (SCON), SE-14104 Huddinge, Sweden; 3 The Swedish Red Cross University College, The Institute of Health Sciences, SE-141 21 Huddinge, Sweden

**Keywords:** Dentistry, Study skills, Theory, Clinical, Undergraduate

## Abstract

**Background:**

The transition from upper secondary to higher education and from higher education to professional practice requires that students adapt to new literacy practices, academic and professional. However, there is a gap of knowledge regarding literacy practices in dental education. Therefore, the aim of this study was to identify what characterizes dental students’ notetaking and secondarily to determine what dental students express regarding their notetaking.

**Methods:**

To analyze students’ perspectives about the purposes of notetaking and to examine their written notes in depth, three volunteer students, out of the 24 students that voluntarily and anonymously handed in their notes, were interviewed. The three undergraduate dental students that participated in this material-based, semi-structured interview study, framed within a New Literacy Studies approach, were on their third year (6th semester). The focus of these material-based interviews was on each student’s notes. Questions prepared for semi-structured interviews were open-ended and allowed for individual follow-up questions related to the interviewee’s answer. To analyze the outcome of the interviews a thematic analysis was used.

**Results:**

From the material-based interviews eight themes that relate to *what*, *how* and *for what purpose* students write were discerned. These eight themes include professional vocabulary, core content as well as clinical examples that belong to *what* students read and write; multimodal accentuation as well as synthesis that belong to *how* students read and write; and mnemonic strategies, academic purposes, and professional purposes that belong to *for what purpose* students read and write.

**Conclusions:**

Findings from the interviews indicate that the digital development, offering a variety of available tools, has expanded the notion of notetaking. This study identified that dental students’ notetaking has changed during their education from initially being synchronous, to also include multimodal and asynchronous writing, making notetaking more of a writing practice. Further, students’ writing practices seem to be motivated by their knowledge formation in relation to a subject matter, but also in relation to their experiences during clinical training. Although, our hypothesis was that the main purpose of notetaking and writing was to pass their course examinations, this study showed that students that were half-way through their dental education, are aware that literacy practices are for learning for their future profession, and not only for passing their exams.

## Background

**Fig. 1:**
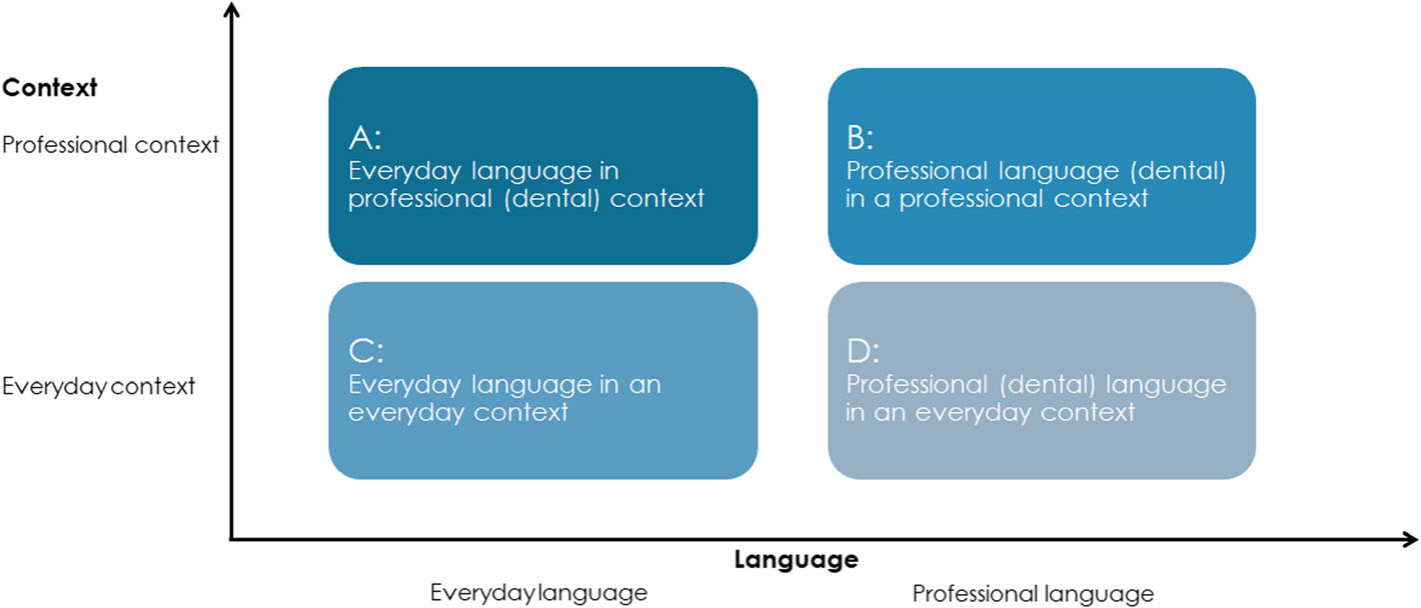
Chart illustrating examples of literacy practices. This chart highlights the relation of context and language (reading, writing, and communicating) in an everyday, an academic and a professional context, modified from Schultz 2001, p. 190 [3]

Literacy is part of learning in higher education where students are expected to read, write and talk about various but new types of texts as compared to previous schooling in various situations. *Literacy practices* is a concept used for defining literacy as situated in a particular context, each characterized by certain ways of making meaning through not only terminology but also by the kinds of texts used and produced, and what qualifies as valid ways of reading, writing and communicating about these texts [1]. For instance, students within study programmes for engineering may read two chapters of a course book for an examination, where the text is used for assignments related to laboratory purposes, while students in study programmes for social work are expected to read approximately 1300 pages for an examination and are expected to summarize thoughts of the writer, analyze particular situations and make conclusions and argue for a decisions regarding a social issue in a specific context. Thus, in professional higher education students have a double task: to learn how to read and write different types of academic texts in ways that are acceptable, i.e. what counts as a good written representation of knowing within a specific context [2, 3], but also to read and write texts used and produced *within* and *for* the profession in context specific acceptable ways. Professional literacy involves not just a specific language unique to a certain profession (e.g. terminology, jargon, and abbreviations), but also specific ways of expressing professional issues, Fig. 1 [4]. Newcomers, in this case students in a dental education, tend to use everyday language before they appropriate the academic language of their education. However, to be able to discern the academic language from the professional language used, is a further step to be taken. The context for language and literacy shifts for students, from every day to education and furthermore to the profession. Over time, students are expected to learn how to shift the way they read, write and communicate in relation to present or absent texts between these contexts. An absent text is a text referred to but not present, such as diagnostic chart [5].

Literacy practices in higher education is a field of research common for researchers from various disciplines, such as anthropology, educational studies, engineering, linguistics, and sociology [6]. Not only *what* you read or write, but also *how* you are expected to read and write, and for *what purposes* you read or write contribute to constructing what counts as valid literacy practices within a specific field, e.g. in this study dental professional literacy. Previous research shows that such literacy practices are socially and historically established, but also develop over time in relation to the scientific and technological development within or for the profession [7]. In a similar way, digital tools also contribute to changing students’ reading and writing practices [8]. Changes in how we interact with various resources (e.g. teachers’ knowledge, textbooks, notes) in various contexts (not only in educational contexts but also in everyday contexts) in combination with the technologies available (smart phones, computers, iPads, apps) “contributes to transforming our conceptions of what learning is” (8; p. 56). Furthermore, societal needs expressed in e.g. regulations that affect a profession also contribute to changes in literacy practices within a profession, which, in turn, affect literacy practices in education programmes [9].

According to Lea and Stierer [3], appropriating new literacy practices requires that students adapt and use new ways of knowing, including new ways of understanding, interpreting, and organizing knowledge. When entering higher education, most students encounter specific academic literacy practices. Studies from a teacher teacher training programme [10], as well as of nursing and engineering programmes [11] show that the transfer, between upper secondary school to academia, has been found challenging and that it takes time to become acquainted with new literacy practices.

Most studies of students’ writing focus assessment situations, and students’ academic texts for assignments and exams are seen “as tangible products of the processes of reading and writing” (( [12]); p. 389) while less attention has been paid to the practices preceding assessment. Such approaches result in an understanding of less successful students’ writing as deficit, and the problem is interpreted as the failing student’s problem, not as part of the teaching practice [13, 14]. Higgs [15] argues that graduates in professional education are required to: **a)** have the abilities to access new information; **b)** judge the information applicability to their specific work setting; **c)** synthesize information from multiple sources (i.e. verbal, printed, and digital); **d)** produce reports and presentations utilizing multiple forms of media; **e)** use information and communication technologies as part of their learning and future professional practice; and **f)** having the ability to critique new information and determine its relevance to a given situation, i.e. in their future profession. The last point **f)** cannot be answered by this study.

In this article, we specifically focus on students writing in one Swedish dental education during a course that starts in the 5th semester of the programme and comprises of four modules spread over four semesters. Reading in Swedish dental education includes textbooks, national guidelines, research articles, PowerPoint-handouts, digital material (in the online-learning environment) such as learning tasks, course instructions and clinical instructions. Writing on the other hand includes notetaking, clinical documentation and tests (Lindberg et al. 2020, submitted).

Thus, to investigate the academic and professional literacy practices of students at an early stage of dental education, this material-based interview study has two aims. The primary aim was to identify what characterizes dental students’ notetaking, and the secondary aim was to determine dental students’ beliefs regarding their notetaking. Our working hypothesis was that dental students perceive that the main purpose of note taking is for passing course examinations.

## Methods

### Context of study

To analyze students’ perspectives about the purposes of notetaking and to examine their written notes in depth, three volunteer students were interviewed in an environment that provided anonymity. These students are referred to as Student 1, 2 and 3. The three students that were interviewed were registered in the module “Orofacial Pain and Jaw Function 2” which is part of the course “Clinical Odontology 2” (https://utbildning.ki.se/course-syllabus/2TL016/24160) in the third year, e.g. 6th semester, of the Study Programme in Dentistry (SPD) at the Department of Dental Medicine at Karolinska Institutet. Since the context of the study has previously been described in detail (Lindberg et al. 2020, submitted), only a summary will follow. At the time of data collection, the total number of full-time students at the SPD was 423, divided into approximately 85 students per academic year. The SPD comprises ten semesters, and five years, providing 300 European Credit Transfer and Accumulation System (ECTS). The first three years are on a basic level and the last two years are on an advanced level. During the course of the SPD, the students are expected to develop in their role as health-care providers, but also to learn about rules and regulations, quality assessments, scientific evaluations, and reflect upon present evidence (https://ki.se/selma/programme-syllabus/2TL13). After graduation, the students apply for licensure from The National Board of Health and Welfare (https://legitimation.socialstyrelsen.se/sv/utbildad-i-sverige/tandlakare) to be allowed to work as dentists in Sweden.

At least three weeks prior to each lecture, teacher-constructed PowerPoints were posted on the online learning platform. During lectures, the students could choose to either write their notes by hand or on digital tools such as a laptops or tablets, on blank sheets or on the PowerPoint-handouts. Altogether, 24 students volunteered to hand over anonymized copies of their notes (in total 237 pages). These were analyzed regarding method used for notetaking, that is, digital and/or paper-and-pencil, and types of notes, which were of three kinds: notes that consist of **1)** copied teacher-written texts; **2)** complemented teacher-written text; or **3)** a mixture of **1** and **2**. After reviewing the notes of these 24 dental students, we asked this cohort for volunteers to participate in in-depth interviews. Since the notes handed over were anonymized, the authors did not know which these 24 students were in the cohort of 68 students registered to the course “Orofacial Pain and Jaw Function 2” (Lindberg et al. 2020 submitted). Therefore, the students were given the possibility to volunteer for participation during March to June 2018. Three students volunteered to participate. The authors do not know the reason why the rest did not volunteer, but possible explanations could be lack of time or just unwillingness to participate in person.

### Material-based interviews

The semi-structured material-based interviews took place on a neutral place for the students, but with academic familiarity. For such interviews, there is an agenda – in this particular case, the focus was on each student’s notes. Questions prepared for semi-structured interviews are open-ended and allow for individual follow-up questions related to the interviewee’s answer [16], starting with questions like “Are these notes representative for the way you take notes in general?” and “Do you have a specific strategy for your notetaking?”. The following questions were related to the content, form and structure of each student’s notes, but also of their purposes with their notes, how they structured them, and how they used them in relation to lectures, other teaching material and examinations [17]. The interviews were conducted in September and October 2018 at Stockholm University by two of the researchers (VL, NCh). Thus, the interviews were not conducted at the Department of Dental Medicine at Karolinska Institutet, which is the place for the dentist programme. The interviews lasted 43.34 min for Student 1, 59.00 min for Student 2, and 114.12 min for Student 3. To secure confidentiality of the interview-setting, that was video recorded, the participants were instructed not to reveal any personal information during the interview. Further, the camera focused only on a table where the students’ notes were displayed, and only the hands of the participants were visible. This because it was important for the researchers to be able to check what aspects of their notes each student emphasized, by pointing at specific parts of their notes [17]. After completion of the interview, the volunteering students left an anonymized copy of their handwritten or digitally written notes from the lectures. These notes consisted of 11 pages from Student 1, 14 pages from Student 2, and 25 pages from Student 3. Further, they also left their digital summaries of the module content consisting of 34 pages from Student 1, 41 pages from Student 2, and 29 pages from Student 3.

This study was based firstly on students’ notes, and secondly on material-based interviews with three of these students. Material-based interviews are basically a theoretical recontextualization of *think-aloud interviews*, originating from cognitivistic traditions, into a New Literacy Studies (NLS) tradition. In her study of teachers’ assessment of nursing students’ written tests, Orrell [18] interviewed teachers while they were reading and assessing students’ responses. The interviews were mainly based on teachers commenting students’ responses aloud. The purpose of Orrell’s study was to highlight experienced teachers’ cognitions during the action of assessment. However, the main idea in think-aloud interviews – to capture the thoughts of the individual interviewees – is problematic for studies that foreground the social practice. The idea that it is possible to claim that you can capture people’s thoughts through interviews has been criticized, firstly based on e.g. Vygotsky’s [19] work. According to him, thinking and speech are related to each other, but they are not the same. Secondly, an interview is a specific kind of conversation: the conditions differ from other kinds of conversations, and interviews also have limitations [20–22]. An interview is a joint construction between interviewer and interviewee: each party have their interpretations of the meaning of the questions posed. Also, the issue of interpretation concerns aspects like what questions are not posed, which answers the interviewer will or will not pose follow-up questions, and what answers the interviewer supports or does not support by various kinds of facial expressions, gestures, humming etc. Most of these aspects are unconscious but can be identified when analyzing interview situations. Based on this critique, interview studies have been methodologically developed to contextualize the interview situation [17, 22]. Since dental students’ notetaking is considered a social practice, we prefer material-based as the concept that describes both the kind of interview we used, but also the theoretical basis for what we claim based on these interviews: the data that are co-constructed.

In the present study the interviewers and interviewees, i.e. students, sat around a table on which the students’ notes were placed, in a small conference room at Stockholm university, which is a familiar academic setting both for the interviewers and the interviewees. Before the actual interview, the students were instructed to speak freely, but to avoid details that could reveal their identity. They were informed that only their answers would be analyzed as well as the notes pointed out by their hands, hence, no facial or body expressions, gestures, confirmatory sounds etc. were to be analyzed. This, to make the interviewees as relaxed as possible. During the interview the students were asked to answer questions regarding their notetaking based on what, how, and why, that is for what purpose. Also, when appropriate, they were asked to highlight their answers with examples from their notes placed on the table. During the course of the interview the interviewers now and then added questions for further clarifications regarding any of the above-mentioned aspects of notetaking.

### Thematic analysis

Thematic analysis may be described as a method for identifying, analyzing, and reporting patterns in a data. Patton [23] and others [24, 25] describe a thematic analytic process as progressing from a description in which data has been organized to reveal patterns of meaning, a summary, an interpretation that attempts to theorize the importance of the patterns, their general meanings and inferences. Thematic analysis is here combined with New Literacy Studies for the purpose of capturing patterned response or meaning within interview data that relates to students’ notetaking. Prevalence is ensured by the refinement of analysis, in themes and potential sub-themes. A theme is determined by what it captures in relation to the research questions. The themes created relate to the data, i.e. the interview transcripts of three students’ notetaking, and were generated by a thorough, inclusive, and comprehensive coding process. Themes include data made sense of, i.e. interpreted, and not data paraphrased or described. Each theme has been described in detail and given a nuanced account.

The analytical procedure involved noticing patterns of meaning and issues of potential interest in the data. Also, analysis involved a relational reading, that is, a constant moving back and forth between the data set, the transcripts analyzed, and the analysis of data produced. The analytical steps performed (by SLJ and MCh) in this study were as follows:

1. The first step concerned familiarization with data which was achieved in the process of transcription of the interviews, that also involved a reading and re-reading of data, and the noting of ideas.
2. In a second step, data for each student was coded in relation to interesting features, which here comprised of the following analytical questions: a) what do they write; b) how do they write; and c) for what purpose do they write?
3. A third step comprised collating codes into preliminary themes, by gathering of relevant data.
4. In a fourth step, these themes were controlled in relation to the previously identified features and in relation to the entire data set. This additionally ensures that themes are internally coherent, consistent and distinctive. The controlling generated a thematic map of the analysis.

1. A fifth step involved the definition of each theme and their naming. The themes (T1-T8; Table 1) were: professional vocabulary, core content, clinical examples, multimodal accentuation, synthesis, mnemonic strategies, academic purposes, and professional purposes. Examples of multimodal accentuation are for instance when students use color for highlighting words or sentences as well as details in pictures, draw arrows between notes in different parts or on teacher-provided handouts.
2. The sixth and final step involved the selection of extract examples that vividly illustrated the themes. These selected extracts were then related back to the research question of the study.

**Table 1:** Overview of themes related to significant analytical questions (AQ) within New Literacy Studies (NLS)

**AQ1: What do students write?**	
*T1: Professional vocabulary*	Concepts and/or descriptions
*T2: Core content*	Teacher accentuated content
*T3: Clinical examples*	Content related to clinical practice
**AQ2: How do students write?**	
*T4: Multimodal accentuation*	Strategies to accentuate content and relations. For instance, color coding, arrows, drawing pictures, pictures and images, upper case letters, tags, visual symbols
*T5: Synthesis*	Students combine PowerPoints from lectures and individual notes, including a presentation comprising a general and detailed structure, usually performed on a computer
**AQ3: Why do students write?**	
*T6: Mnemonic strategies*	Knowledge and understanding
*T7: Academic purposes*	Exam-preparation
*T8: Professional purposes*	Professional knowledge and support; **a)** clinical skills as student and dentist **b)** the development of professional literacy

## Results

Results from this study showed that students usually write different types of texts (Lindberg et al., 2020, submitted) for the development of a complete understanding of the subject matter. Students’ writing is motivated by the development of knowledge and understanding of a subject matter, but also for study achievement and future professional development and support.

Based on the analytical questions from NLS and the thematic analysis (Table 1) the following themes (T1–8) were discerned based upon the questions: what do students write, how do students write, and for what purpose do students write?

### What do students write?

Three recurrent themes were identified in relation to what students write. The students wrote complementary text that comprised of: *T1)* professional vocabulary; *T2)* core content; and *T3)* clinical examples. Complementary text included information exceeding what was presented in the PowerPoints. For instance, complementary text were teacher examples that related to the content in the PowerPoint, for instance clinical examples that vocationally or clinically contextualized teaching and learning content. A specific example was Student 3 that noted a simile provided by the teacher, where Posselt’s trajectory was compared to the shape of a banana. Student 3 argued that additional information provided by the teacher during lectures, supports memorizing, and is therefore noteworthy.

Professional vocabulary, i.e. concepts and descriptions, involved the simplification or description of terminology or other phenomena in accordance with students’ individual and everyday language. For instance, Student 3 added the verb ‘movement’ for further describing ‘exaggerated protrusion of the mandibula’. The same student also added ‘goes back and forth’ to describe the terms ‘protrusion’ and ‘retrusion’.

All three students discerned core content, i.e. central, recurrent and accentuated (e.g. intonation) content by the teachers during lectures. An example from all three students was the word ‘toxic’, another was ‘pain’ and ‘peripheral sensitization’, accentuated by the teachers. Another example described by the three students was the repetition that patient recollection was a significant source of information for treatment.

**Fig. 2:**
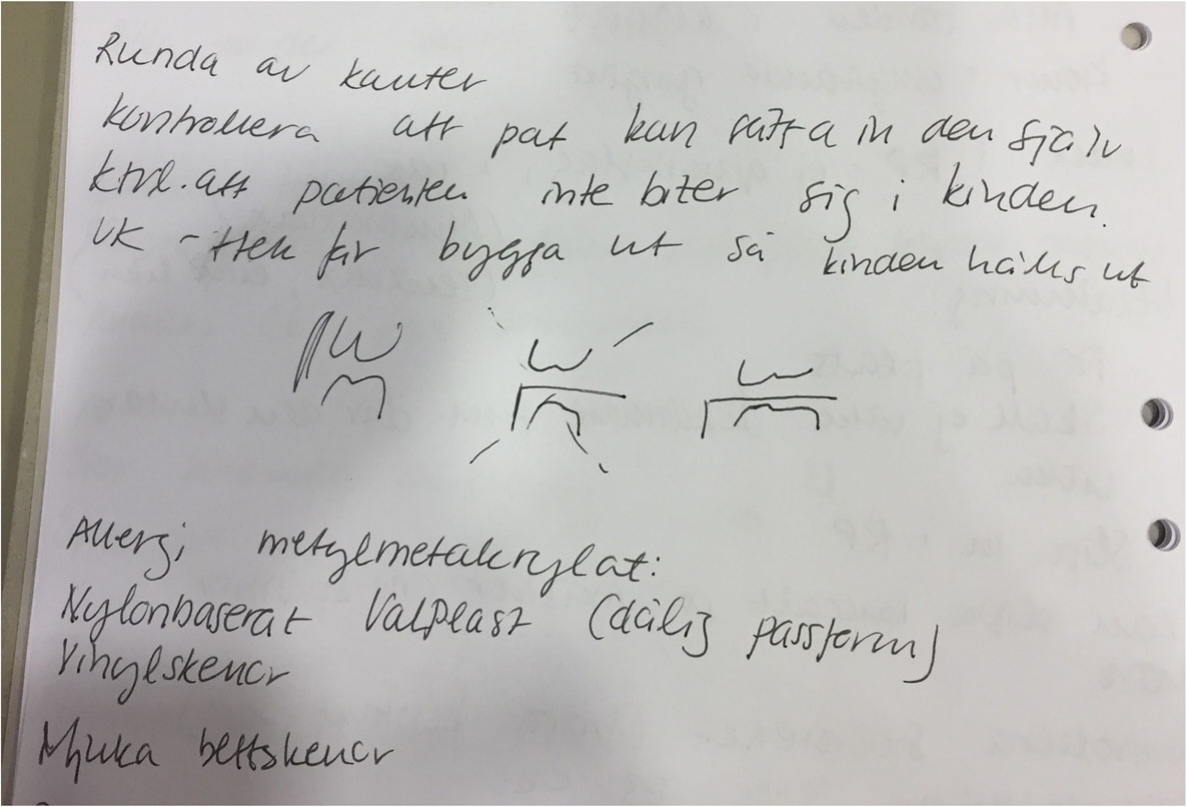
Photocopy of student 2 handwritten notetaking. This shows notetaking for professional purposes. The notes have a clinically relevant content regarding the adjustment and delivery of an occlusal appliance, a common treatment for orofacial pain conditions. In the middle of the note, the student added teacher-examples by copying the teacher’s drawings from the whiteboard

Clinically relevant content was provided to students based on teachers’ experiences, i.e. examples from their clinical practices. Teacher-examples were occasionally further supported by drawings on the whiteboard. Students copied these drawings into their own notes (Fig. 2).

### How do students write?

**Fig. 3:**
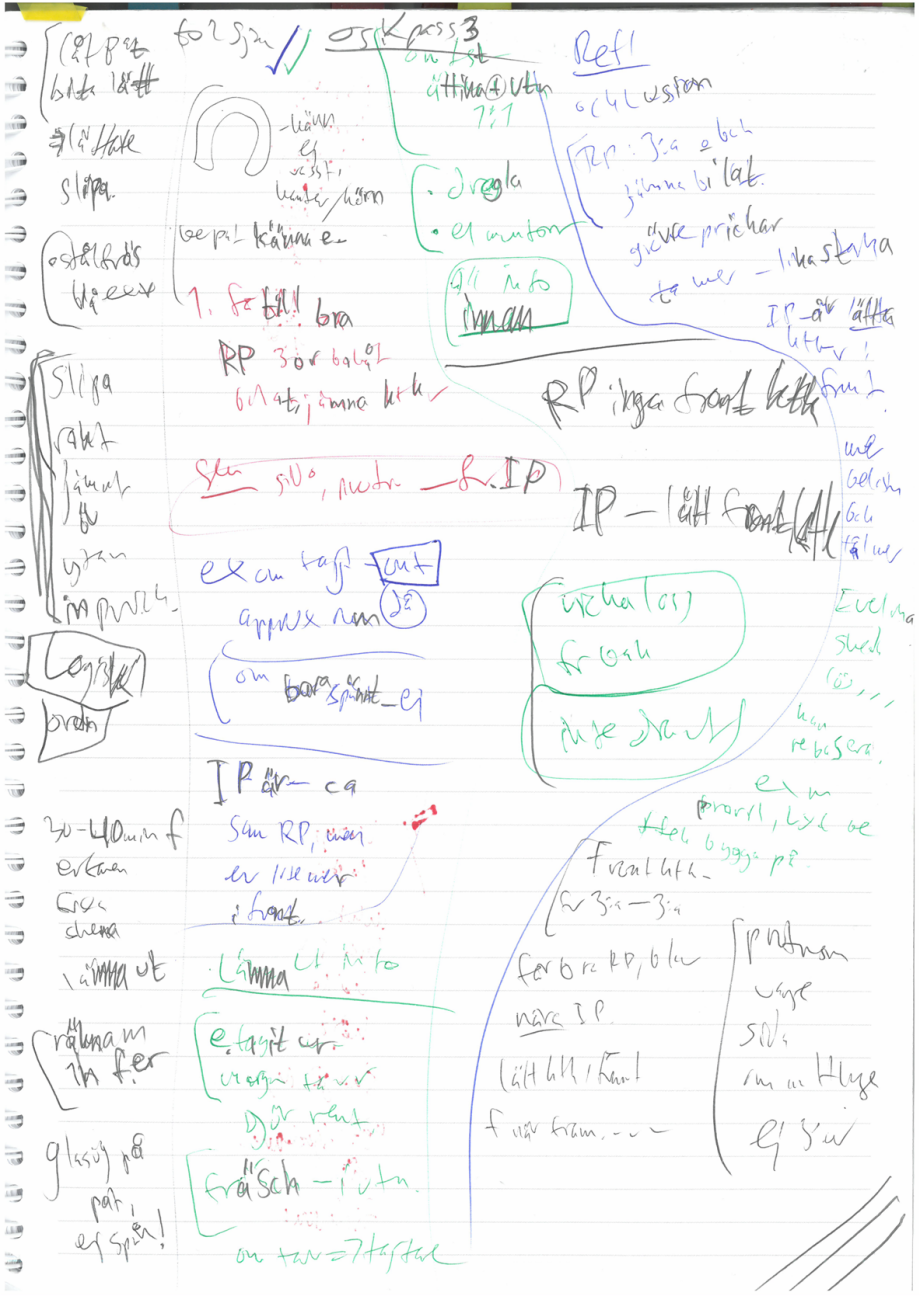
Photocopy of student 1 handwritten notetaking. Student 1 uses black color as basic color for notetaking, and three to four other colors in order to signify different things. The colors were then used to group information about the same topic, for instance blue to clarify, green for professional purposes, and red to highlight important clinical aspects

Two themes were discerned in relation to how students write, and these were: *T4)* multimodal strategies; and *T5)* syntheses. Multimodal strategies that students used aimed to support memory, and to aid their understanding of the subject. Strategies comprised of various color coding; accentuation through different types of markings, such as squares, bolding and exclamation marks; arrows marking movement or leading to various descriptions; drawing own pictures, or adding pictures and images for further explanation of content in text; upper case letters; tags; visual symbols for instance marking female and male gender. For instance, Students 1 and 3 used black color as basic color for noting, while other colors signified different things (Fig. 3). Also, Student 2 used red color for marking words, concepts or other information that needs to be further controlled and thus clarified as an adequate explanation that was not provided in the lecture itself. After the lecture, all three students either looked up in books, searched the internet or called a course-friend to ask. Student 3 also used yellow highlight to mark something important, while pink highlight to mark concepts and terminology. Further, both Students 2 and 3 expressed that their notetaking had changed over time. From just being synchronous handwritten notes on blank papers or on printed handouts based on the teacher-constructed PowerPoints, to asynchronous and multimodal. This is illustrated by an excerpt from the interview with Student 3 (interview conducted 2018-09-25, authors’ translation):

I have used notebooks for long time and think it works well resulting in good results in exams, but people around me uses computers and tablets. But sometimes I could not read my notes, and it goes faster with the computer. What I am lacking with my handwritten notes are the coloring, but there are other advantages with the computer. / … / so I have changed my way of writing notes.

**Fig. 4:**
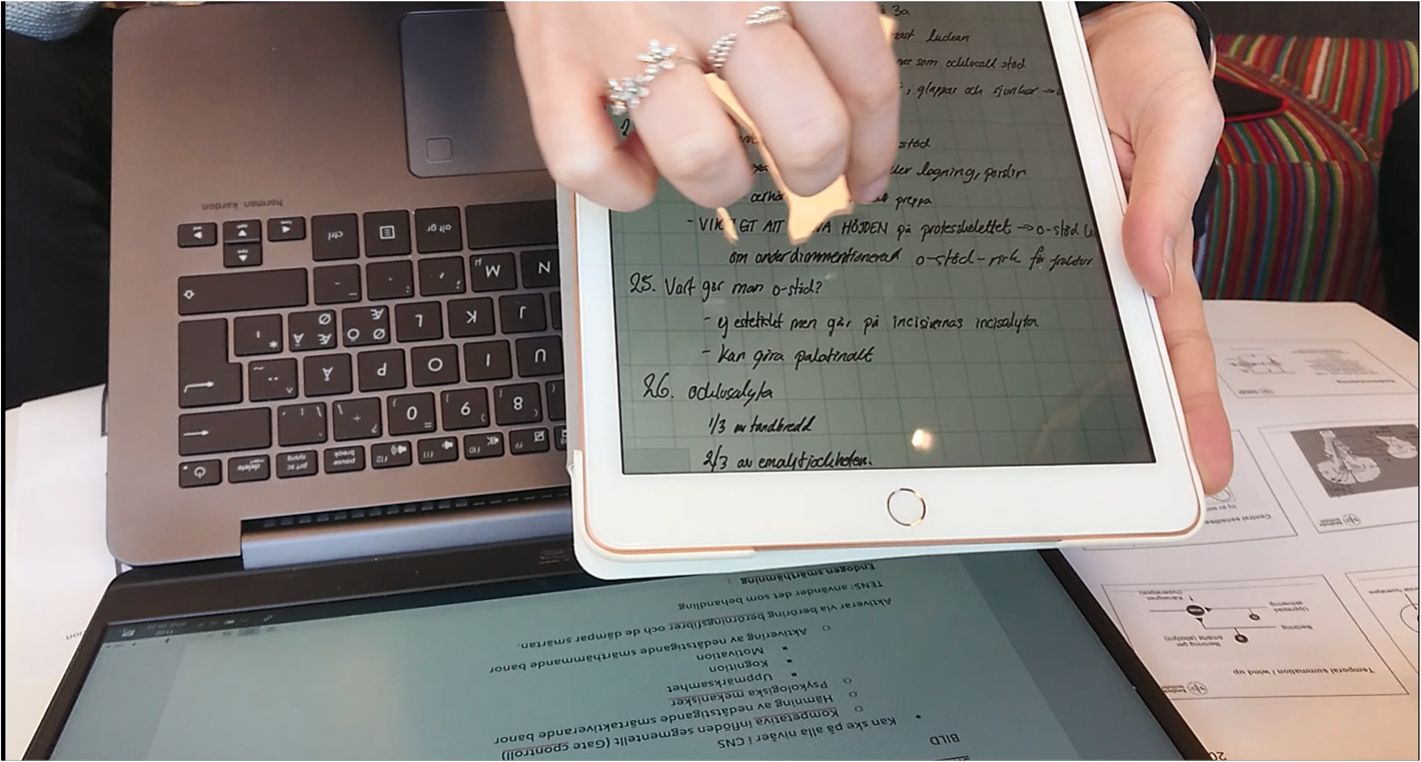
Photograph displaying student 3 demonstrating a multimodal combination of notetaking. Notetaking is based on **a**) PowerPoint-handouts from lectures at the lower right part of photograph; **b**) teacher information from lectures summarized in a Word-document in the computer screen, at the lower border of the photograph; and **c**) individual complementary handwritten notes on the tablet, in the middle of the photograph, that refers to the specific page at the PowerPoint-handout, seen at the lower right part of photograph

Student 3’s synthesis comprised of a multimodal combination of PowerPoint-handouts from lectures, additional teacher information from lectures summarized in Word-documents, and students’ individual complementary handwritten notes on tablets, referring to specific page on the PowerPoint-handout (Fig. 4). All three students often complemented the notes with a synthesis soon after the lectures, as a mnemonic strategy to increase the chance of remembering details from the lecture. The student synthesis was a structured summary of the subject, including general and detailed information. These syntheses helped the students to reach a broader and deeper understanding of the subject.

### Why do students write?

Three themes were identified for the question of why students write: *T6)* mnemonic strategies; *T7)* academic purposes; and *T8)* professional purposes (Fig. 2). Students notetaking as a mnemonic strategy, was presented by all three students as clarifications of vocabulary, examinations, diagnoses as well as procedures, and as helping the three students to relate different information to each other. Thus, the three students connected different parts of a subject to create a whole understanding of the content.

Also, all three students used notetaking and constructed syntheses to pass their exams and thus explicitly to achieve the learning objectives set for the educational programme. According to all three students, notetaking was an essential part of preparing for examinations. They emphasized that notetaking required understanding of what was noted and understanding of how and why things were connected within a subject.

In terms of professional purposes, the three students interviewed expressed an awareness of literacy-related demands in the dentist profession. Thus, they all considered reading different types of texts and writing as part of a professional development and highly relevant as preparation for their future profession as dentists.

## Discussion

This study shows that the technical advancement with the digital tools that were provided on the online learning environment as well as those tools, such as laptops and tablets, that the students chose to bring to the lectures, resulted in developed literacy practices. In relation to text-genres we found that students’ notes related to three genres: **a)** the dental academic language needed for exams and presentation with teachers as the receiving part, i.e. anatomy, physiology and pathology/diagnoses related to textbooks and teachers’ handouts, that also is the basis for their future professional terminology; **b)** professionally specific language in national and international guidelines for dentistry as well as diagnostic protocols to be used as tools for professional judgement and diagnosis; and **c)** professionally specific language to be used in clinical contexts with other dentists, caregivers and patients as receiving parties. Explicit for this last genre is that these professional texts are to be written but they are often also used as a basis for oral communication with the mentioned receiving parties. In comparison to Dias and colleagues [11] the three literacy genres the dental students needed to appropriate, within the frame of the studied modules, seem to be more in agreement with professional demands, than Dias and colleagues [11] found for nursing and engineering students. However, a point to be made, is that it is also expected that some of these dental students will have a future career in academia.

In relation to Berthén and colleagues [13] our findings confirm that most of the notetaking strategies are relevant for higher education and future professional practice: the interviewed students tried **1)** to identify the main focus of lectures; **2)** took notes related to what the teachers said; and **3)** added notes about their own ideas of important issues related to the purpose of the lecture, which were based on what was emphasized during the lecture or by the teachers’ clinical experiences. Furthermore, they wrote complementary texts for mnemonic purposes and posed questions for clarification to the teachers during as well as after lectures. The students also summarized their notes afterwards including their own thoughts or questions to teachers. This represents a kind of personalized synthesis of the core content – not only of the teachers’ words, which is valued as an indication of higher-order cognitive knowing [26]. However, none of the interviewed students deliberately compared their notes with those of fellow students, but they expressed that they occasionally borrowed notes or asked another student if they found something unclear. All three students also copied teachers’ PowerPoints to some extent, but copying was complemented with other strategies.

A strength of the study is the large amount of student notes (237 pages) handed in, including all modalities used by Swedish dental students (handwritten, digital, illustrations). Therefore, this study was able to capture a wide perspective of an unexplored area concerning literacy practices in dental education. A second strength is the combination of analytical procedures, including both student notes and in-depth semi-structured material-based interviews. On the other hand, there are some concerns. Studies on dropouts from higher education [27] show that the response rate from less successful students, even in questionnaires, is quite low. Our assumption is that these students’ notes represent firstly successful students’ notes, and secondly that Swedish is their native language. This can be seen as a methodological problem, since less successful students and students with Swedish as second language can be surmised to hesitate to share their notes.

## Conclusion

This study aimed to identify the characteristics of dental students’ note writing, and secondarily, to determine students’ perceptions about the purposes and strategies of their notetaking. Findings from the interviews indicate that the digital development, offering a variety of available tools, has expanded the notion of notetaking. Thus, this study identified that dental students’ notetaking has changed during their education from initially just being synchronous, to now also include multimodal and asynchronous writing, making the notetaking more of a writing practice. Secondly, the students expressed that they posed questions for clarification to the teachers during as well as after lectures and wrote complementary texts for mnemonic purposes, to be able to summarize their notes afterwards in pamphlets including their own thoughts or answers to their questions to teachers. This represents a kind of personalized synthesis of the core content. Thus, the students’ writing practices seem to be motivated by their knowledge formation in relation to a subject matter, but also in relation to their experiences during clinical training. Finally, our hypothesis was not confirmed, instead, this study showed that students, half-way through their dental education, are aware of that literacy practices are for learning for their future profession, and not only for passing their exams.

Take-home message: **1)** teachers can use communicative strategies, i.e. how to talk about content in texts, for the development of a professional language as well as notes for professional purposes; **2)** the technical advancement with the digital tools that are provided has resulted in evolved literacy practices, e.g. multimodal notes; **3)** when planning new courses the course designers should take into account the digital literacy opportunities provided; and **4)** course designers are recommended to analyze their students’ literacy practices and take them into account when designing new courses or improving existing courses.

## Data Availability

The materials used and analyzed during the current study are available from the corresponding author on reasonable request.

## Abbreviations

ECTS: European Credit Transfer and Accumulation System
NLS: New Literacy Studies
SPD: Study Programme in Dentistry
